# Therapeutic potential of ginseng leaf extract in inhibiting mast cell-mediated allergic inflammation and atopic dermatitis-like skin inflammation in DNCB-treated mice

**DOI:** 10.3389/fphar.2024.1403285

**Published:** 2024-05-22

**Authors:** Jung-Mi Oh, HyunHo Yoon, Jae-Yeol Joo, Wan-Taek Im, Sungkun Chun

**Affiliations:** ^1^ Department of Physiology, Institute for Medical Sciences, Jeonbuk National University Medical School, Jeonju, Jeollabuk-do, Republic of Korea; ^2^ Department of Pharmacy, College of Pharmacy, Hanyang University, Ansan, Gyeonggi-do, Republic of Korea; ^3^ Department of Biological Sciences, Hankyong National University, Anseong, Gyeonggi-do, Republic of Korea

**Keywords:** ginseng leaf extract, inflammasome, pro-inflammatory cytokines, degranulation, MAPK, NF-κB, anti-inflammatory

## Abstract

Ginseng leaves are known to contain high concentrations of bioactive compounds, such as ginsenosides, and have potential as a treatment for various conditions, including fungal infections, cancer, obesity, oxidative stress, and age-related diseases. This study assessed the impact of ginseng leaf extract (GLE) on mast cell-mediated allergic inflammation and atopic dermatitis (AD) in DNCB-treated mice. GLE reduced skin thickness and lymph node nodules and suppressed the expression and secretion of histamine and pro-inflammatory cytokines. It also significantly lowered the production of inflammatory response mediators including ROS, leukotriene C4 (LTC4), prostaglandin E2 (PGE2), cyclooxygenase-2 (COX-2), and inducible nitric oxide synthase (iNOS). GLE inhibited the phosphorylation of MAPKs (ERK, P38, JNK) and the activation of NF-κB, which are both linked to inflammatory cytokine expression. We demonstrated that GLE’s inhibitory effect on mast cell-mediated allergic inflammation is due to the blockade of the NF-κB and inflammasome pathways. Our findings suggest that GLE can be an effective therapeutic agent for mast-cell mediated and allergic inflammatory conditions.

## Graphical Abstract

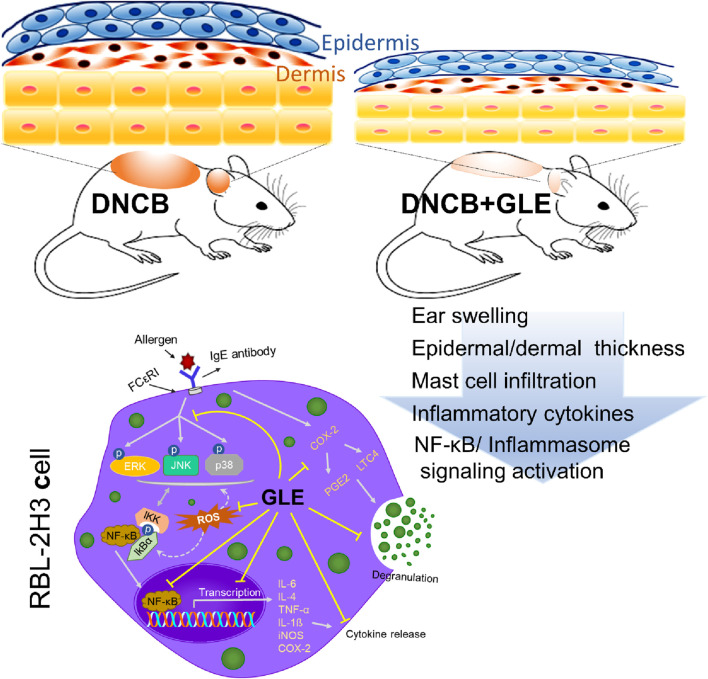

## Highlights

● Topical application of GLE improves symptoms of DNCB-induced atopic dermatitis.
● GLE treatment suppressed the production of inflammatory cytokines and mediators in DNCB-AD mice.
● GLE inhibits inflammasome and NF-κB signaling in DNCB-AD mice and MAPK/NF-κB signaling in activated mast cells.
● GLE can suppress the production of IgE and ROS as well as the expression of inflammatory cytokines and mediators in activated mast cells.

## 1 Introduction

Atopic dermatitis (AD) is the most prevalent chronic skin condition and has multiple causes, including genetic predisposition, disruption of the epidermal barrier, and dysregulation of the immune system (Kim H. et al., 2014). AD is characterized by increased serum levels of total immunoglobulin E (IgE), epidermal thickening, impaired skin barriers, and infiltration of inflammatory cells (i.e., lymphocytes, macrophages, eosinophils, and mast cells) (Kim J. et al., 2019). Among them, mast cells are major mediators of IgE-mediated allergic disorders, including allergic rhinitis, asthma, and atopic dermatitis (Galli and Tsai, 2010; Burton and Oettgen, 2011; Galli and Tsai, 2012). The typical physiological pathway for mast cell activation is the cross-linking of Fc portion of the ε heavy chain on the mast cell’s IgE receptor (FcεRI) with allergens (Sibilano et al., 2014). Activated mast cells release various biological mediators, such as histamines, inflammatory cytokines, chemokines, eicosanoids, lipid mediators, and vasoconstrictor compounds, which can trigger allergic inflammatory responses. Histamine released from activated mast cells triggers acute allergic reactions. Arachidonic acid metabolites, such as LTB4 and LTC4, attract neutrophils and trigger late-phase allergic reactions (Gilfillan and Tkaczyk, 2006; Ogawa and Calhoun, 2006). Cytokines such as IL-4, IL-5 and IL-6 secreted by Th2 cells stimulate B cells to increase IgE production, respectively, contributing to mast cell activation and allergic inflammation (Solimando et al., 2022). During the late stage response of allergic inflammation, mast cells release proinflammatory mediators such as tumor necrosis factor (TNF)-α, interleukin (IL)-4, IL-1β, and IL-8 (Ye et al., 2017). Mediators produced in this way increase IgE levels and trigger the degranulation of mast cells, ultimately contributing to chronic allergic reactions (Brandt and Sivaprasad, 2011; Langan et al., 2020). To alleviate symptoms of allergic inflammation, it is important to decrease the levels of histamine and pro-inflammatory cytokines. Activation of FcεRI by IgE/Ag-mediated cross-linking leads to the activation of Src family kinases (Fyn and Lyn), which initiates mast cell degranulation. This, in turn, triggers intracellular signal cascades including MAPK, NF-κB, and phospholipase C γ (PLCγ) pathways (Osorio-Perez et al., 2023).The intracellular signaling cascade induced by IgE–FcεRI aggregation in mast cells has been extensively studied (Krystel-Whittemore et al., 2015). Many studies have shown that inflammatory substances secreted by mast cells play an important role in inducing and maintaining allergic reactions. For this reason, controlling the differentiation or activation of mast cells would be a useful target for treatment to reduce allergic reactions.

Inflammatory innate immune responses are essential in host defenses against pathogens. However, dysregulated and excessive inflammatory reactions lead to tissue damage and inflammation and are regarded as an underlying cause of some human diseases and disorders such as AD (Vanaja et al., 2015; Broz and Dixit, 2016; Wang and Hauenstein, 2020). Among the major inflammatory pathways in these diseases is the activation of inflammasomes (Chen and Nunez, 2011; Prochnicki and Latz, 2017).

Inflammasomes contribute to host defenses against infection and internal danger signals by promoting the secretion of pro-inflammatory cytokines IL-1β and IL-18 and inducing pyroptosis (Prochnicki and Latz, 2017). Among the nucleotide oligomerization domain (NOD)-like receptors (NLR), the NLRP3 inflammasome is the most studied. NLRP3 is a multiprotein complex composed of the NLRP3 scaffold, an adaptor for apoptosis-associated speck-like protein (ASC), and the effector pro-caspase-1 (casp-1). NLRP3 inflammasome formation is initiated by the assembly of ASC and pro-casp-1, which leads to the cleavage and activation of pro-casp-1, IL-1β, and IL-18, resulting in the secretion of bioactive IL-1β and IL-18 (Lu et al., 2014; Jimenez-Duran and Triantafilou, 2021; Triantafilou, 2021). Numerous studies have confirmed the involvement of the inflammasome in various diseases, such as psoriasis, vitiligo, systemic lupus erythematosus, and AD (de Koning et al., 2012; Douglas et al., 2015; Hiramoto et al., 2018; Tang and Zhou, 2020). Moreover, external triggers of atopy, such as exposure to house dust mites and *staphylococcus aureus* infection, have been found to active the inflammasome complex (Ogawa et al., 2008; Munoz-Planillo et al., 2009; Jacquet, 2011a; Jacquet, 2011b; Dai et al., 2011; Niebuhr et al., 2014; Ruxrungtham, 2016). This finding suggests that the inflammasome induce AD and that inhibiting NLRP3 inflammasomes be an effective therapeutic strategy for AD.

Therapeutic agents, such as topical and oral corticosteroids, topical calcineurin inhibitors, anti-histamines, and immunosuppressants, are currently used to treat AD (He et al., 2018a; He et al., 2018b), but the prolonged use of these medications can have various adverse effects (D'Auria et al., 2016; Czarnowicki et al., 2017). New, safe long-term treatment strategies are therefore required, and natural compounds are currently attracting attention as an alternative to existing medicines. Ginseng has been used as an herbal medicine for millennia for its antiallergy (Han and Kim, 2020), anticancer (Oh et al., 2019a; Oh et al., 2019b; Hong et al., 2021), antidiabetic (Attele et al., 2002; Chen et al., 2019), and anti-inflammatory (Im, 2020) effects. Korean red ginseng has been reported to effectively suppress atopic skin inflammation and improve skin (Hong and Lyu, 2011; Park et al., 2019). Furthermore, ginsenosides, which are the active ingredients responsible for the pharmacological effects of ginseng, have been reported to have various effects, including anti-allergy and immunomodulatory activities (Kee and Hong, 2019; Valdes-Gonzalez et al., 2023). Even though the strong effects of ginsenosides were already provided, the disadvantages of them are likely that most of the ginsenosides are extracted from ginseng roots, which poses economic constraints due to their high cost for therapeutic use compared to leaves and stems (Ceylan-Isik et al., 2008; Wang et al., 2009).

Recent studies have reported that ginseng leaves also contain highly concentrated, physiologically active ingredients, including ginsenosides, polyacetylene alcohol, amino and fatty acids, polysaccharides, triterpenoids, and flavonoids (Hou, 1977; Zhang et al., 2008; Wang et al., 2009). American ginseng (*Panax quinquefolius*) leaves have been found to contain significantly higher concentrations of ginsenoside components than its roots and berries (Xie et al., 2004). It has been demonstrated that GLE has positive pharmacological effects on the central nervous, cardiovascular, and immune systems (Wang et al., 2009). However, it is not clear whether GLE has an anti-allergic effect on mast cells, and there are few studies on its therapeutic and anti-allergic effect on AD. Thus, we explored the impact of GLE on anti-allergic inflammation and its underlying molecular mechanism in this study.

## 2 Material and methods

### 2.1 Reagents and antibodies

Dulbecco’s Modified Eagle Medium (DMEM), penicillin-streptomycin, 0.05% Trypsin-EDTA, and fetal bovine serum (FBS) were purchased from GIBCO (Grand Island, NY, United States of America). 3-(4,5-dimethylthiazol-2-yl)-2,5-diphenyltetrazoliumbromide (MTT) was obtained from Molecular Probes (Eugene, OR, United States of America). Anti-dinitrophenyl-(DNP) IgE (monoclonal anti-dinitrophenyl antibody produced in mouse, IgE isotype, SPE-7 clone), DNP-bovine serum albumin (BSA), 4-nitrophenyl N-acetyl-β-D-glucosaminide (p-NAG), 3-(4,5-dimethylthiazol-2-yl)-2, dimethyl sulfoxide (DMSO), 2′,7′dichlorodihydrofluorescein diacetate (DCFH-DA), and phosphatidylinositol 3 (PI3) kinase selective inhibitor (LY294002) were obtained from Sigma-Aldrich (St. Louis, MO, United States of America). A Griess reagent system for nitric oxide (NO) measurements was purchased from Promega (Madison, WI, United States of America).

We used primary antibodies against phosphor (p)-P38 (#9211), P38 (#9212), p-ERK1/2 (#9101), ERK1/2 (#4696), p-JNK (#9255), JNK(#9252), Ac-NF-κB (#12629), casp1 (#24232), NLRP3 (#15101), IL-1ß (#31202), ASC (#67824), AIM2 (#63660), and cyclooxygenase-2 (COX-2; #12282), as well as mouse (#7076) or rabbit (#7074)-horseradish peroxidase (HRP)-conjugated secondary antibodies; all were purchased from Cell Signaling Technology (Beverly, MA, United States of America). Specific antibodies against p-IκBα (sc-8404), Lamin B (sc-6216) were obtained from Santa Cruz Biotechnology (Dallas, TX, United States of America), and NF-κB (ab1602) and TNF-α (ab6671) from Abcam (Cambridge, United Kingdom). Glyceralde-hyde-3-phosphate dehydrogenase (GAPDH; AP0063) was purchased from Bioworld Technology Inc. (Bloomington, MN, United States of America).

### 2.2 Preparation of American GLE

One kg of American ginseng leaf powder was obtained by double extraction with 20 L of 70% ethanol. The extract was filtered through paper and dried using a rotary evaporator. The resulting dried powder was dissolved in water and loaded onto a glass column (400 mm L × 100 mm D) packed with Diaion HP-20 resin (Mitsubishi Chemical, Tokyo, Japan). Free sugar molecules and unwanted hydrophilic compounds from HP-20-absorbed beads were washed with eight-column volumes of water, and the attached ginsenosides were eluted with six-column volumes of 80% ethanol. The ethanol extracts were evaporated in a vacuum, and the dried residue was used for the following cell tests.

According to a high-performance liquid chromatography (HPLC) analysis, the extract was mainly composed of Rg1 (9.4 mg/g), Re (61.1 mg/g), Rb1 (9.8 mg/g), Rb2, (41.2 mg/g), Rb3 (175 mg/g), Rc (16.2 mg/g), and Rd (94.7 mg/g); it also contained small amounts of other ginsenosides (Supplementary Figure S1). The GLE was dissolved in 50 mM acetate buffer and solubilized up to 50 mg/mL. HPLC-grade methanol and acetonitrile were obtained from SK Chemical Co., Ltd. (Seoul, Republic of Korea). The GLE extract underwent low-temperature filtration and evaporation before being freeze-dried, powdered, and stored at −70 °C until use. All other chemicals used in this study were of an analytical or higher grade. The HPLC analysis was conducted as previously described (Siddiqi et al., 2021).

### 2.3 Cell culture and cell viability assay

The RBL-2H3 cell line was kindly provided by Prof. Seung-Hyun Kim (Ajou University, Suwon, Republic of Korea). The cells were incubated in DMEM supplemented with 10% FBS and antibiotics (100 U/mL penicillin and 100 μg/mL streptomycin) at 37°C in a humidified 5% CO_2_ atmosphere (Thermo Fisher Scientific, Waltham, MA, United States of America). Cell viability was tested using an MTT assay. RBL-2H3 cells (4 × 10^4^) were transferred into 96-well plates (Corning, AZ, United States of America) and incubated for 24 h at 37 °C in different GLE concentrations (10, 20, 40, 80, and 100 μg/mL). After the medium was discarded, the cells were incubated for another 3 h at 37 °C with fresh medium containing 0.5 mg/mL MTT. The formazan products were dissolved in 200 µL DMSO and measured at 570 nm using the Synergy™ 2 microplate reader (BioTek Instruments Inc., Winooski, VT, United States of America). Cell viability was expressed as a percentage of the control, which was set to 100. All experiments were performed in triplicate and repeated at least three times.

### 2.4 Mast cell degranulation assay

To determine the amount of β-hexosaminidase or histamine released in the supernatant, an RBL-2H3 degranulation assay was performed as previously described (Yang et al., 2018). In brief, RBL-2H3 cells were incubated in 24-well plates (1 × 10^5^ cells/well) at 37 °C overnight. The cells were washed with 4-(2-hydroxyethyl)-1-piperazineethanesulfonic acid (HEPES)-buffered saline (140 mM NaCl, 5 mM KCl, 1 mM CaCl_2_, 0.6 mM MgCl_2_, 0.1% glucose, 0.1% BSA, and 10 mM HEPES) and then incubated with anti-DNP-IgE (100 ng/mL) for 24 h at 37 °C. IgE-sensitized cells were pre-incubated with GLE (0, 10, 20, 40 μg/mL) or the positive control, dexamethasone (10 μΜ) for 1 h. Subsequently, 200 ng/mL DNP-BSA was added and incubated for 2 h. The cell culture medium was centrifuged (17,000 ×g, 10 min) at 4 °C, and the supernatant was recovered.

To determine β-hexosaminidase levels, the supernatant was transferred to a 96-well plate (50 µL/well). Fifty µL substrate solution was added (1 mM p-nitrophenyl-N-acetyl-β-D-glucosaminide (p-NAG) in 100 mM citrate, pH 4.5) and left to react at 37 °C for 3 h. The reaction was terminated with a stop buffer (0.1 M Na2CO_3_-NaHCO_3_, pH 10.0), and the absorbance was determined at 405 nm using the Synergy™2 microplate reader (BioTek Instruments Inc., Winooski, VT, United States of America). The supernatant was also used to measure the release of histamine, according to the manufacturer’s protocol for the enzyme immunoassay system (Oxford Biomedical Research, Rochester Hills, MI, United States of America). Data are expressed as the mean ± standard deviation (SD) of triplicate experiments, and the results represent the percentage changes from the controls.

The percentage of β-hexosaminidase or histamine release was calculated using the following formula (Yang et al., 2013): β-hexosaminidase or histamine release (%) = [(T-B-N)/(C-N)] x100; T (test): DNP-BSA (+), GLE (+); B (blank): DNP-BSA (−), GLE (+); C (control): DNP-BSA (+), GLE (−); N (normal): DNP-BSA (−), GLE (−).

### 2.5 Measurement of intracellular ROS and NO

RBL-2H3 cells were suspended in DMEM containing 10% FBS and then dispensed to a volume of 2 × 10^4^ cells in a 96-well plate (Corning, AZ, United States of America); then anti-DNP-IgE (100 ng/mL) was added, and the solution was incubated for 24 h in a 5% CO_2_ incubator at 37 °C.

IgE-sensitized cells were incubated with 10 μM H_2_DCF-DA at 37 °C for 30 min. The residual H_2_DCF-DA in the medium was removed with PBS. The cells were treated with GLE (0, 20, 40 μg/mL), Dexa (10 μM), and 10 mM N-acetylcysteine (NAC) for 1 h. Subsequently, the cells were stimulated with DNP-BSA (200 ng/mL) for 30 min. For the image analysis, the cells were washed with cold PBS and then imaged under a CELENA^®^ S fluorescence microscope (Logos Biosystems, Inc., Anyang, Republic of Korea). Three microscope fields on average were quantified in three separate cultures per treatment condition, and ImageJ software (NIH, Bethesda, MA, United States of America) was used to quantify fluorescence intensity. The accumulated nitrite in the cell supernatant was measured using the Griess reagent system (Promega, Madison, WI, United States of America) according to the manufacturer’s instructions.

### 2.6 Measurement of cytokine secretion

Anti-DNP-IgE-sensitized RBL-2H3 cells were pretreated with GLE (0, 20, 40 μg/mL) for 1 h and then stimulated with DNP-BSA (200 ng/mL) and incubated for 2 h. To measure the TNF-α, IL-6, IL-1ß, IL-4, PGE2, and LTC4 concentrations in the culture media (cell supernatants), all samples were centrifuged (17,000 ×g, 10 min) at 4 °C and stored at −80 °C until analyzed. The various concentrations of cytokines were measured using ELISA kits according to each manufacturer’s instructions. Enzyme immunoassay kits for prostaglandin E2 (PGE2) and leukotriene C4 (LTC4) were purchased from Cayman Chemical (Ann Arbor, MI, United States of America). The ELISA kits for TNF-α, IL-6, IL-1ß, and IL-4 were obtained from KOMA Biotech (Seoul, Republic of Korea). Data are expressed as mean ± SD of the triplicate experiments. Cytokine levels were normalized using a protein assay kit (BD Biosciences, CA, United States of America) and calculated as picograms per milligram (pg/mg) of the total protein.

### 2.7 RT-PCR

To evaluate the effect of GLE on pro-inflammatory gene expression, RBL-2H3 cells were seeded in a 6-well plate at a volume of 3 × 10^5^ cells. The cells sensitized with anti-DNP-IgE (100 ng/mL) were pre-treated with GLE or Dexa for 1 h and then challenged with DNP-BSA (200 ng/mL) for 2 h. The total RNA of cells was extracted with TRIzol^®^ reagent (Invitrogen, Carlsbad, CA, United States of America), according to the manufacturer’s protocol, and 5 µg of RNA was used for complementary DNA (cDNA) synthesis with the GoScript reverse transcription system (Promega, Madison, WI, United States of America). PCR was carried out with specific primers. The PCR products were electrophoresed on a 1.2% agarose gel and visualized on a UV transilluminator with RedSafe™ (iNtRON Biotechnology, Seongnam, Republic of Korea). The relative expression level of each target gene was quantified by normalization to the internal GAPDH control gene. The primers used in the experiment are listed in Table 1.

**TABLE 1:** Primer sequences used for RT-PCR analysis.

Genes	Forward primer (5′-3′)	Reverse primer (5′-3′)
*Gapdh*	CTC​GTG​GAG​TCT​ACT​GGT​GT	GTC​ATC​ATA​CTT​GGC​AGG​TT
*IL-4*	ATG​GGT​CTC​AAC​CCC​CAG​CTA​GT	GCT​CTT​TAC​GCT​TTC​CAG​GAA​GTC
*IL-6*	AGT TGC CTT CTT GGGACT GAT	TCC ACG ATT TCC CAG AGA AC
*IL-1* β	ATG GCA ACT GTT CCT GAA CTC AAC	ATA TTC TGT CCA TTG AGG TGG AGA GCT
*iNOS*	TGG GAA TGG AGA CTG TCC CAG	ACC GCCT TCT GGT CGA TGT CA
*TNF-* α	AGC CCC CAG TCT GTA TCC TT	CTC CCTTTG CAG AAC TCA GG
*COX-2*	CCC CCA CAG TCA AAG ACA CT	TTC TGC AGC CAT TTC CTT CT

IL, interleukin; RT-PCR, reverse transcription polymerase chain reaction; TNF, tumor necrosis factor; COX-2, cyclooxygenase-2; iNOS, inducible nitric oxide synthase, GAPDH, glyceraldehyde 3-phosphate dehydrogenase.

### 2.8 Western blot analysis

Anti-DNP-IgE-sensitized RBL-2H3 cells were pretreated with GLE (0, 40 μg/mL) or Dexa (10 μM) for 1 h and then stimulated with DNP-BSA (200 ng/mL) for 30 min (MAPK) and (TNF-α, COX-2, p-IκBα, and NF-κB) incubated for 1 h. The supernatant was removed, cells were washed twice with ice-cold PBS. The nuclei and cytoplasmic proteins were extracted using a nuclear extraction kit (Abcam, Cambridge, MA, United States of America) following the manufacturer’s instructions. Whole cell extracts were lysed with radioimmunoprecipitation assay (RIPA) buffer (Thermo Fisher Scientific, Waltham, MA, United States of America), and mouse ear skin tissue were lysed with T-PER (Thermo Fisher Scientific). Halt protease and phosphatase inhibitor cocktails (Thermo Fisher Scientific, Waltham, MA, United States of America) was added to these two buffer solutions. The cell lysates were separated by 12% sodium dodecyl sulfate-polyacrylamide gel electrophoresis (SDS-PAGE) and transferred onto a polyvinylidene fluoride (PVDF) membrane (Millipore, Burlington, MA, United States of America). The membrane was blocked with 5% skim milk or 5% BSA for 1 h at room temperature, then incubated overnight with primary antibodies in a 1:1000 dilution. The blots were washed with Tris-buffered saline with 0.1% Tween^®^ 20 Detergent (TBST) and then incubated with HRP-conjugated IgG secondary antibodies (1:5,000) for 1 h at room temperature. Protein band intensity was measured using an Amersham Imager 600 (GE Healthcare, Piscataway, NJ, United States of America). The target protein concentrations were compared to the controls, and the density of each band was determined using ImageJ software.

### 2.9 Animals

Six-week-old male BALB/c mice were purchased from the Nara Bio Animal Center (Nara Biotech, Seoul, Republic of Korea). These experiments were approved by the Jeonbuk National University Institutional Animal Care and Use Committee (approval no. NON 2023-199). Before beginning the experiment, the mice had a 1-week acclimatization period. Food and water were provided *ad libitum*, and the animals were kept in a controlled environment, with a temperature of 23°C ± 3 °C, a relative humidity between 40% and 60%, and a 12-hour light and dark cycle. The mice were randomly divided into four groups (n = 5 per group): (I) the control (non-treated) group, (II) the 2,4-dinitrochlorobenzene (DNCB) + vehicle group, (III) the DNCB +50 mg/kg GLE group, (IV) the DNCB +100 mg/kg GLE group, and (V) DNCB +100 mg/kg Dexamethasone (Dexa).

### 2.10 Allergic dermatitis model and treatment protocols

AD-like skin lesions were induced by applying a DNCB solution Initially, the mice’s backs and ears were shaved and administered with 200 μL of 1% DNCB in a mixture of acetone and olive oil (4:1) over a period of 3 days (Days 1–3). After 7 days, 0.5% DNCB was applied to their backs and ears three times a week (Days 7–35) for 4 weeks. GLE treatment was applied at concentrations of 50 and 100 mg/kg in a 200 μL mixture of saline and olive oil (9:1) every other day for 35 days. On Day 35, all mice were euthanized (Figure 6). For dexamethasone (positive control) group, the mice were intraperitoneally (IP) injected with 1 mg/kg of dexamethasone for three times a week. The skin of their ear lesions was removed for Western blot, ELISA, and histopathological analysis.

### 2.11 Histological analysis

The ears of the mice were excised and fixed in 10% neutral buffered formalin for 24 h. After fixation, the skin was embedded in paraffin and sectioned at a thickness of 5 μm. The thickness of the ear skin dermis and epidermis was measured through H&E staining. Toluidine blue staining was used to measure the number of mast cells. All stained tissues were examined by light microscopy (Leica, IL, United States of America), using the Leica Application Suite microscope software (Leica Microsystems Inc., IL, United States of America). Mast cell counts in 0.025 mm^2^ samples were obtained by conducting assessments at ×200 magnification and analyzing the resulting images using ImageJ software.

### 2.12 Blood analysis and measurement of biochemical analysis

Blood samples were collected through cardiac puncture and transferred into EDTA-containing tubes (BD Science, NJ, United States of America). The samples were centrifuged at 2000 rpm for 10 min at 4 °C. The serum was collected and kept at −80 °C until analysis. The levels of histamine (Oxford Biomedical Research, United States of America), PGE2 (Cayman Chemical, United States of America), IgE, IL-6, TNF-α, and IL-4 (KOMA Biotech, Republic of Korea) in the mouse serum were assessed using ELISA kits according to the manufacturer’s recommendation.

### 2.13 Statistical analysis

All data were analyzed using GraphPad Prism software 10 (GraphPad Software, Inc., La Jolla, CA, United States of America). Group differences between mean values of normally distributed data were analyzed and assessed using one-way analysis of variance (ANOVA) followed by Duncan’s test for multiple comparisons. All experimental results are expressed as mean ± SD or mean ± SEM, and values of *p* < 0.05, *p* < 0.01, and *p* < 0.001 were considered statistically significant.

## 3 Results

### 3.1 GLE treatment inhibits the degranulation of DNP-IgE/BSA-sensitized RBL-2H3 cells

**FIGURE 1:**
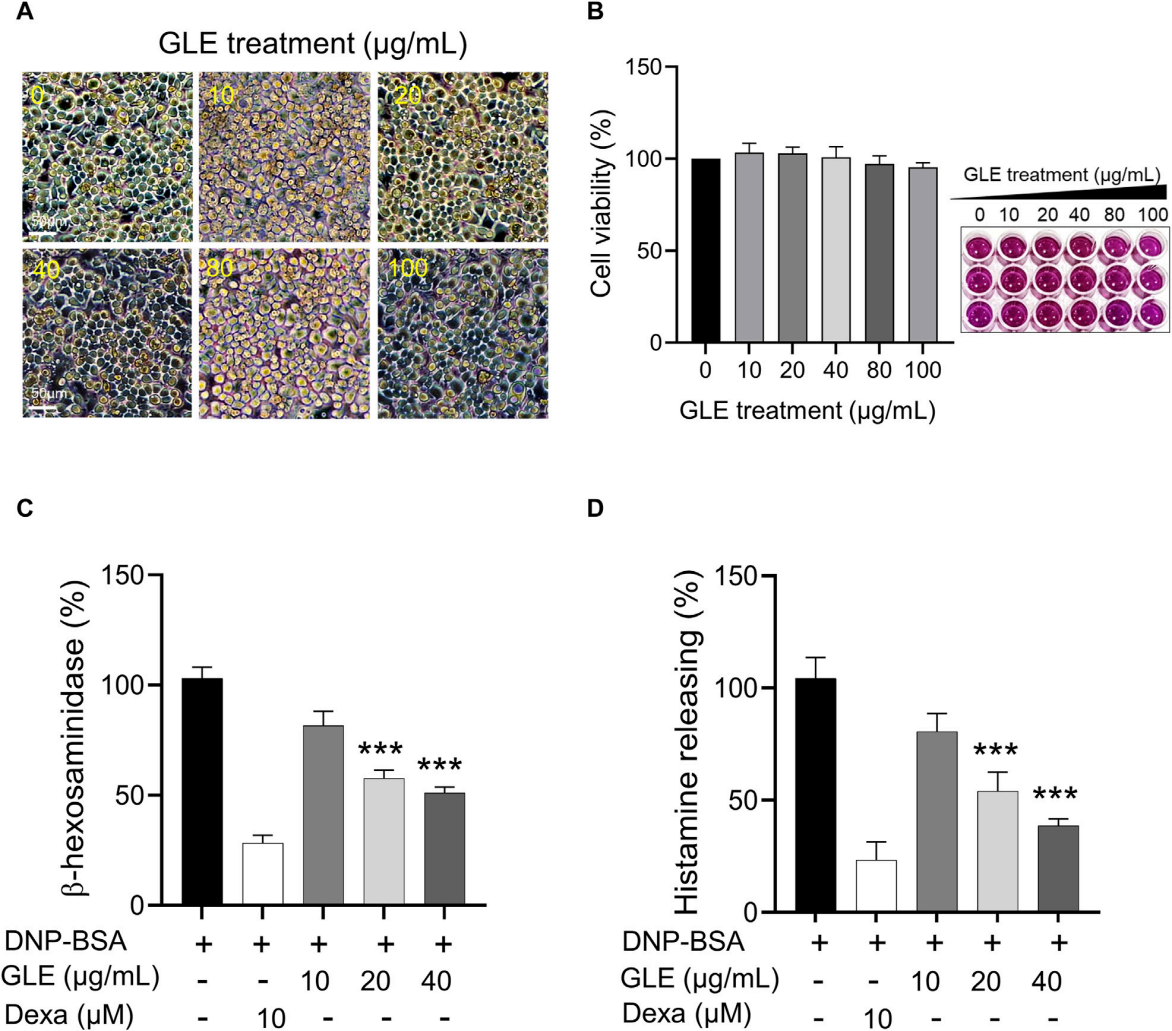
Inhibition of β-hexosaminiase and histamine release in RBL-2H3 cells with GLE treatment. **(A, B)** An MTT assay was performed to determine the cell viability of RBL-2H3 cells treated with different concentrations of GLE (0–100 μg/mL) for 24 h. The effect of GLE treatment on cell morphology was visualized under a microscope, with a scale bar of 50 µm. To induce mast cell degranulation, RBL-2H3 cells (1 × 10^5^ cells/well) were sensitized with anti-DNP IgE (100 ng/mL) for 24 h. The cells that had been sensitized were pre-treated with GLE or Dexa for 1 h and then stimulated with DNP-BSA (200 ng/mL) for 2 h. The amount of ß-hexosaminidase was quantified using ß-hexosaminidase substrate buffer **(C)**, and the quantity of histamine was dettermined utilizing an ELISA kit **(D)**. The mean ± SD of three independent experiments was used to express the data. Statistical significance is indicated as *p < 0.05, **p < 0.01, and ***p < 0.001, compared to the DNP-BSA-treated group. ELISA: enzyme-linked immunosorbent assay, SD:standard deviation, Dexa: dexamethasone, GLE: ginseng leaf extract.

To assess the impact of GLE on mast cell growth, unstimulated RBL-2H3 cells (without IgE/DNP-BSA treatment) were treated with various concentrations of GLE (0–100 μg/mL) and subjected to MTT assays. As depicted in Figure 1A, B, treatment with GLE up to a concentration of 100 μg/mL did not result in any signs of cytotoxicity. To analyze GLE’s effect on RBL-2H3 cell degranulation, we measured the production of histamine and ß-hexosaminidase following DNP-IgE/BSA stimulation and administered the dexamethasone as a positive control. Our findings indicated that the release of histamine and β-hexosaminidase, which was enhanced by DNP-IgE/BSA activation, was reduced in a concentration-dependent manner by GLE treatment (20 and 40 μg/mL) (Figure 1C, D).

### 3.2 GLE inhibits the mRNA expression and secretion of pro-inflammatory cytokines in DNP-IgE/BSA-sensitized RBL-2H3 cells

**FIGURE 2:**
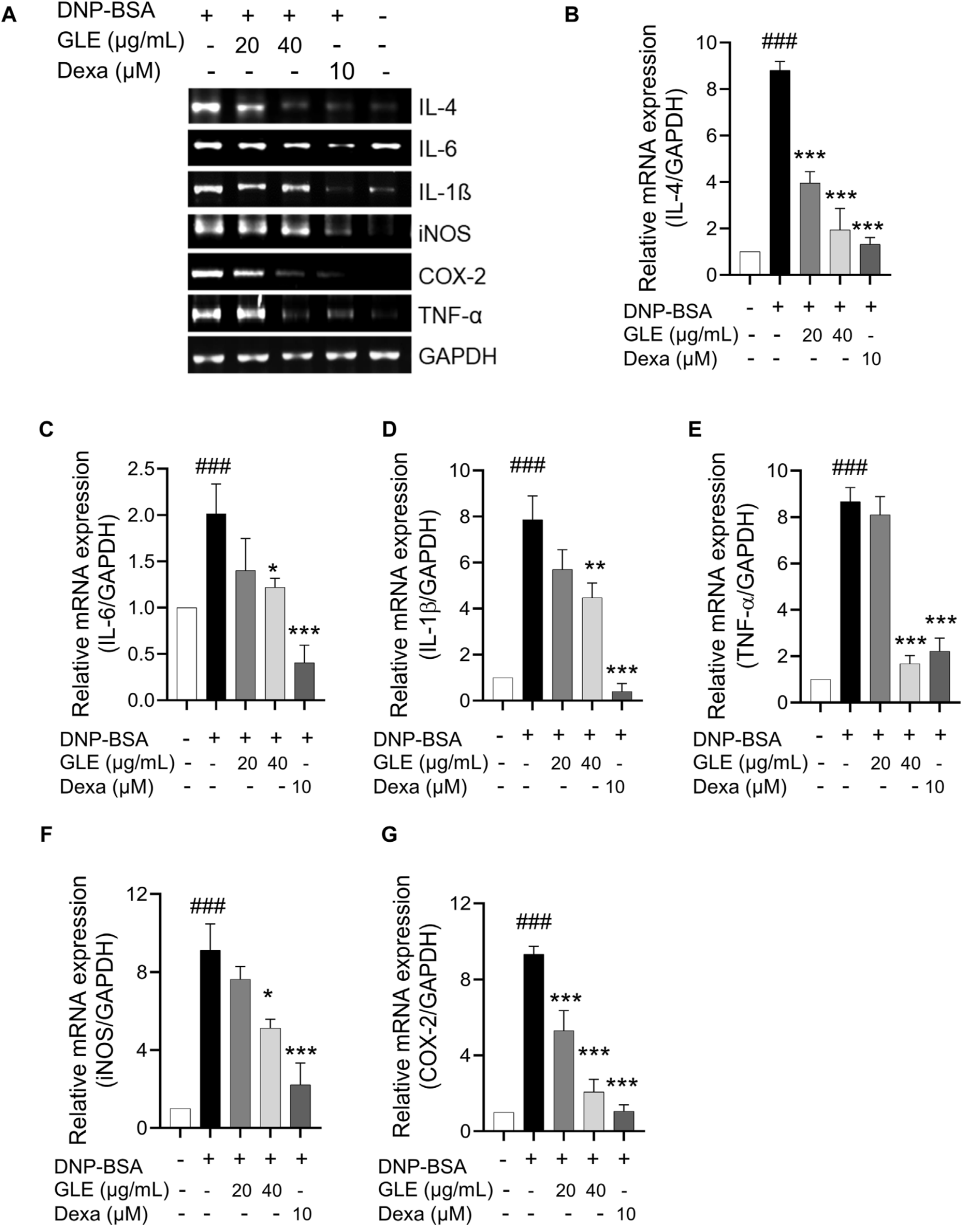
In IgE-activated RBL-2H3 cells, the expression of IL-4, IL-6, TNF-α, IL-1ß, iNOS and COX-2 is inhibited by GLE. After 24 h of sensitization with DNP-IgE (100 ng/mL), RBL-2H3 cells were subjected to a 1-h treatment with GLE (20 and 40 µM) followed by exposure to DNP-BSA (200 ng/mL) for 2 h. The mRNA expression levels of IL-4 **(A, B)**, IL-6 **(A, C)**, IL-1ß **(A, D)**, TNF-α **(A, E)**, iNOS **(A, F)** and COX-2 **(A, G)** were assessed using semi-quantitative reverse transcription polymerase chain reaction (RT-PCR). An internal control, glyceraldehyde-3-phosphate dehydrogenase (GAPDH), was utilized. **(B–F)** Densitometry analysis using ImageJ software was employed to determine the band intensities. The data, presented as mean ± SD, were obtained from a minimum of three independent experiments. The notation ^###^ indicates statistical significance at *p* < 0.001 compared to the control group, while * represents *p* < 0.05 and *** denotes *p* < 0.001 compared to the DNP/IgE + BSA-activated group. The control group refers to the untreated group, IL: interleukin, COX-2: cyclooxygenase-2, iNOS: inducible nitric oxide synthase, TNF-α: tumor necrosis factor, DNP-BSA: anti-dinitrophenyl-bovine serum albumin, Dexa: dexamethason.

**FIGURE 3:**
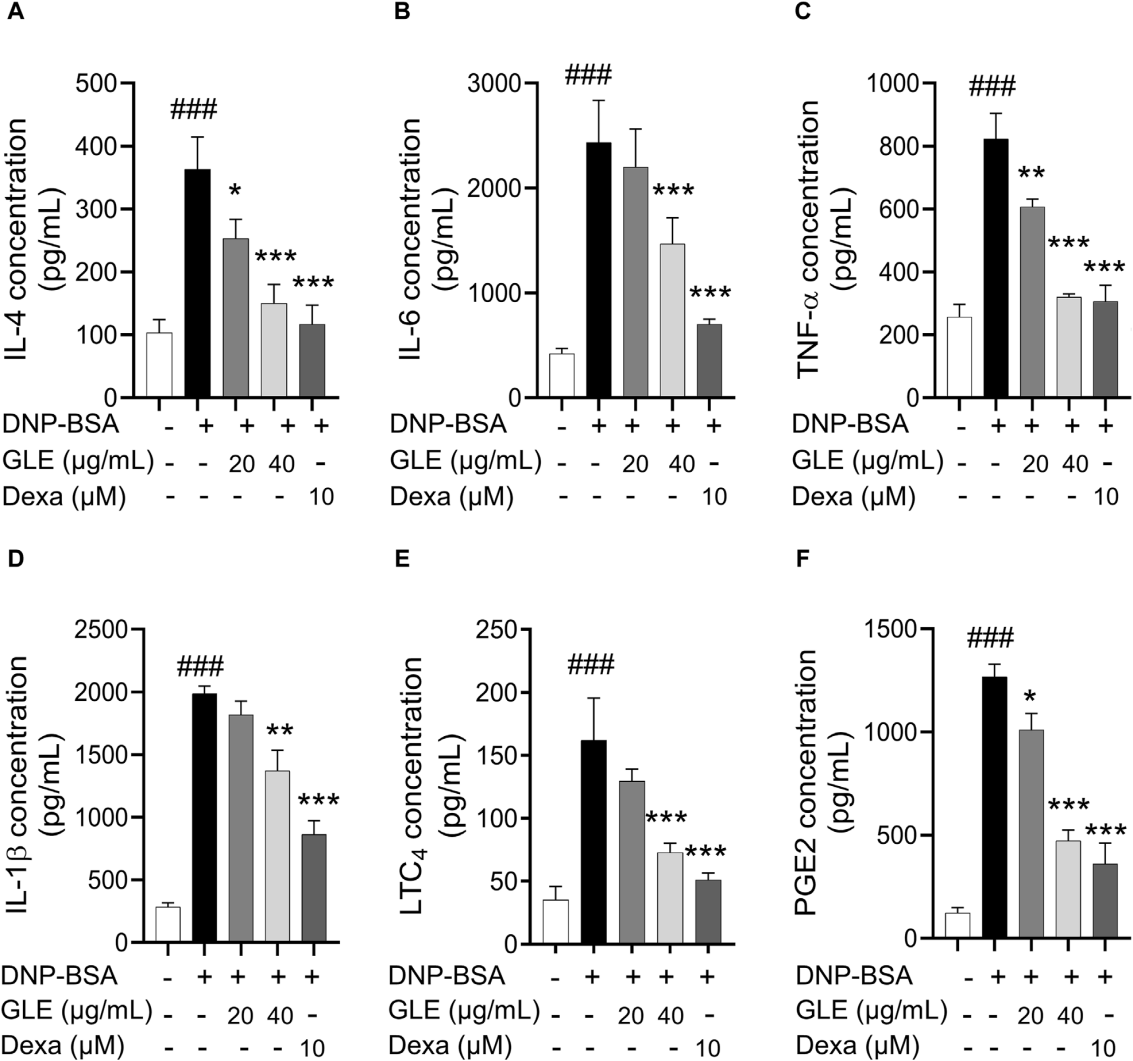
In IgE-activated RBL-2H3 cells, GLE effectively suppresses the secretion of pro-inflammatory cytokines IL-4, IL-6, IL-1ß, TNF-α, PGE2 and LTC4. Prior to the 2 h stimulation with DNP-BSA, IgE-sensitized RBL-2H3 cells were treated with GLE (20 and 40 µM) or Dexa (10 µM) for 1 h before stimulation with DNP-BSA for 1 h. The levels of pro-inflammatory cytokines IL-4 **(A)**, IL-6 **(B)**, TNF-α **(C)**, IL-1ß **(D)**, LTC4 **(E)** and PGE2 **(F)** in the supernatants were quantified using an ELISA assay. The results are expressed as mean ± SD of three independent experiments. The statistical significance is denoted as follows: ^###^: *p* < 0.001 compared to the control group, *: *p* < 0.05, **: *p* < 0.01, ***: *p* < 0.001 compared to the IgE/DNP-BSA-activated group. The control group refers to the untreated group. DNP-BSA: anti-dinitrophenyl-bovine serum albumin, GLE: ginseng leaf extract, IL: interleukin, TNF-α: tumor necrosis factor-α, LTC4: leukotriene C4, PGE2: prostaglandin E2, Dexa: dexamethasone.

To ascertain GLE’s anti-inflammatory characteristics, we investigated the mRNA expression and secretion levels of pro-inflammatory cytokines in IgE/DNP-BSA stimulated RBL-2H3 cells using RT-PCR (Figure 2) and ELISA (Figure 3) methods. The findings demonstrated that GLE treatment significantly reduced the mRNA expression and secretion levels of IL-4, IL-6, IL-1β, TNF-α, which were increased by IgE/DNP-BSA treatment (Figure 2A–E; Figure 3A–D). GLE treatment also significantly suppressed iNOS and COX-2 mRNA expression (Figure 2F, G) and decreased the secretion levels of both LTC4 and PGE2 (Figure 3E, F) compared to the IgE/DNP-BSA stimulation group. The binding of FcεRI to IgE-activated mast cells is associated with the activation of the arachidonate cascade (Gilfillan and Tkaczyk, 2006). Our results indicate that GLE inhibits the enzymes involved in the biosynthesis of prostaglandin and leukotriene, significantly suppressing the formation of LTC4 and PGE2 in IgE/DNP-BSA-stimulated RBL-2H3 cells. Collectively, these results suggest that GLE treatment of IgE/DNP-BSA-stimulated RBL-2H3 cells dose-dependently suppress the expression and release of pro-inflammatory cytokines.

### 3.3 GLE reduces ROS production in DNP-IgE/BSA-sensitized RBL-2H3 cells

**FIGURE 4:**
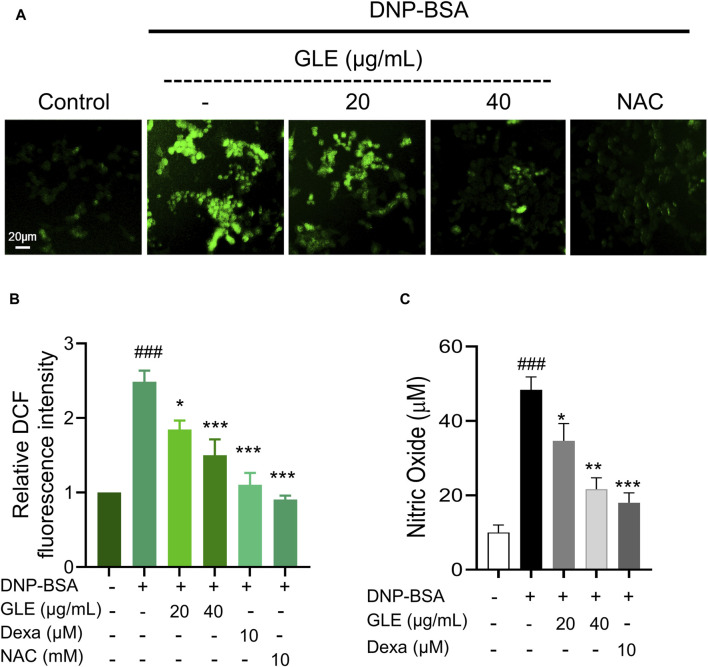
GLE effectively suppresses the production of reactive oxygen species (ROS) in IgE/DNP + BSA-activated RBL-2H3 cells. RBL-2H3 cells were seeded at a density of 2 × 10^4^ cells per well in 96-well plates and sensitized with anti-DNP-IgE (100 ng/mL) for 24 h. IgE-stimulated RBL-2H3 cells were pretreated with 10 µM H_2_DCF-DA and incubated with GLE, Dexa, and NAC for 1 h. Subsequently, they were treated with DNP-BSA (200 ng/mL) for 30 min. **(A)** Fluorescence microscopy was utilized to measure ROS generation (magnification ×10). **(B)** A histogram of the relative DCF intensities was created using ImageJ software. **(C)** The measurement of nitric oxide release was conducted using the Griess Reagent System. The data in **(B)** and **(C)** are presented as mean ± SD. Statistical significance is denoted as ### for *p* < 0.001 vs. control group, ** for *p* < 0.01, and *** for *p* < 0.001 vs. the IgE/DNP + BSA-activated group. The control group refers to the untreated group. DNP-BSA: anti-dinitrophenyl-bovine serum albumin, GLE: ginseng leaf extract, DCFH-DA: 2′,7′-dichlorodihydrofluorescein diacetate, NAC: N-acetyl-l-cysteine, Dexa: dexamethasone.

ROS is reportedly essential to oxidative and inflammatory responses (Nakai et al., 2012; Ji and Li, 2016). To confirm the impact of GLE on ROS production in IgE/DNP-BSA-activated mast cells, we employed DCFH-DA, a reducing fluorescence indicator that can be oxidized by H_2_O_2_. Fluorescence microscopic examination revealed that the intracellular ROS levels increased by IgE/DNP-BSA activation in RBL-2H3 cells were significantly reduced by GLE treatment (Figures 4A, B). The production of NO in IgE/DNP-BSA-activated RBL-2H3 cells also notably decreased following GLE treatment (Figure 4C). Together, these results indicate that GLE treatment effectively suppressed the elevated ROS and NO levels by antigen stimulation in RBL-2H3 cells.

### 3.4 GLE inhibits activation of MAPK and NF-κB in DNP-IgE/BSA-sensitized RBL-2H3 cells

**FIGURE 5:**
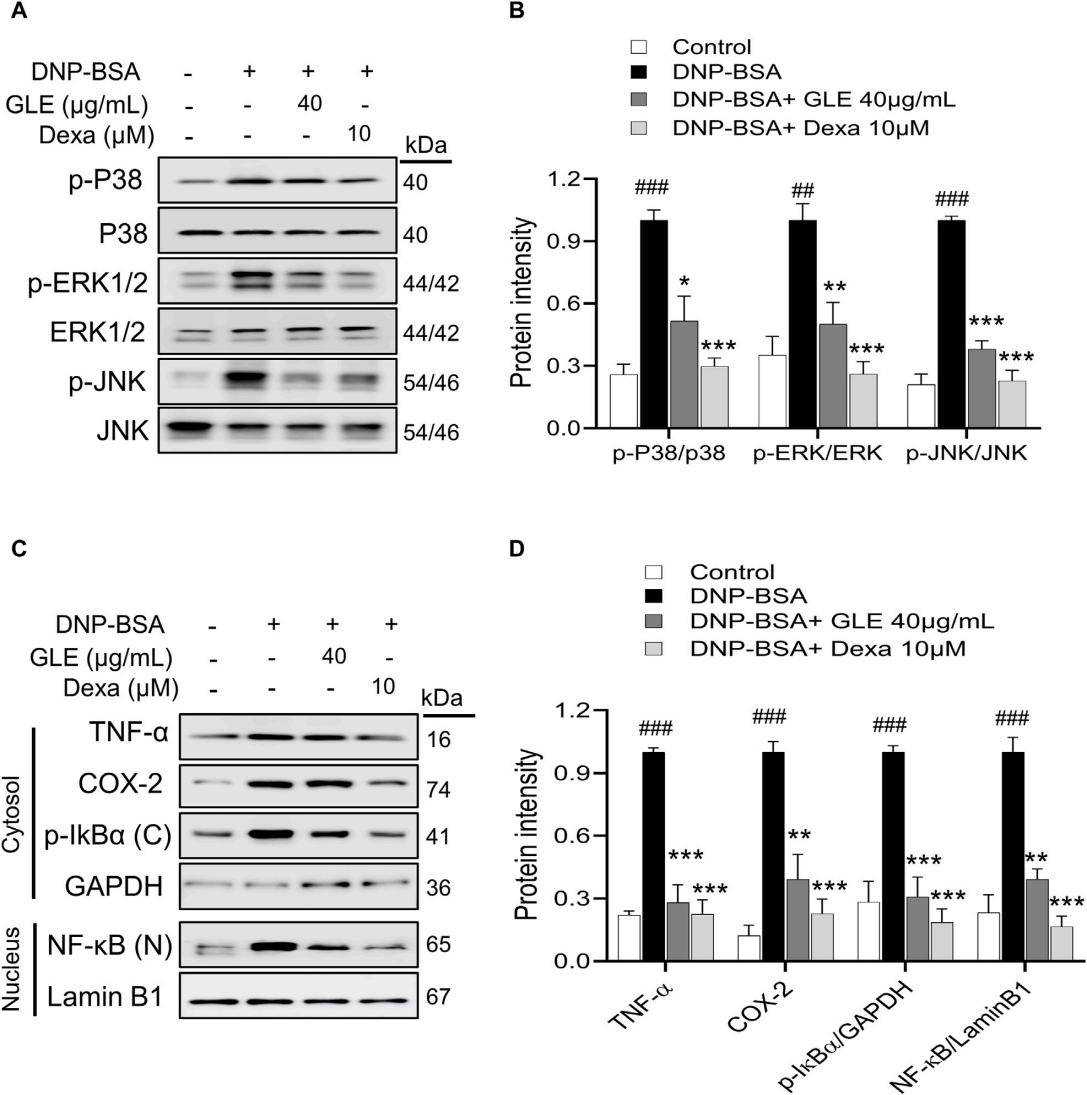
In IgE-activated RBL-2H3 cells, GLE suppresses the phosphorylation of MAPK, prevents the degradation of IκBα and inhibits the translocation of NF-κB. RBL-2H3 cells were seeded at a density of 3 × 10^5^ cells per well in six-well plates and then sensitized with anti-DNP-IgE (100 ng/mL) for a duration of 24 h. IgE-sensitized to RBL-2H3 cells were treated with GLE (40 µM) and Dexa (10 µM) for 1 h, followed by DNP-BSA (200 ng/mL) for 30 min **(A, B)** and 1 h **(C, D)**. **(A, B)** Phosphorylation of p-P38, p-ERK1/2 and p-JNK was assessed using Western blotting. Relative ratios of total protein, determined through densitometry analyses, were employed as loading controls. **(C, D)** The nuclear (N) and cytoplasmic **(C)** proteins were separated using 12% SDS-PAGE gels and analyzed through Western blotting with anti- NF-κB and p-IκBα antibodies. Western blotting was performed to detect the expression of cytosolic p-IκBα and nuclear NF-κB protein. The cytosol **(C)** and nuclear (N) loading controls were determined using GAPDH and Lamin B1, respectively. Densitometry analysis using ImageJ software was used to determine the band intensities in **(B)** and **(D)**. The data, presented as mean ± SD, were obtained from at least three independent experiments. ### indicates *p* < 0.001 compared to the control group, * denotes *p* < 0.05, ** represents *p* < 0.1 and *** signifies *p* < 0.001 compared to the IgE/DNP + BSA-activated group. The control group refers to the untreated group. DNP-BSA: anti-dinitrophenyl-bovine serum albumin, TNF-α: tumor necrosis factor-α, COX-2: cyclooxygenase-2, NF-κB: nuclear factor kappa-light-chain-enhancer of activated B cell, IκB: NF kappa-B-inhibitor alpha, GLE: ginseng leaf extract, Dexa: dexamethasone.

MAPK phosphorylation has been linked to inflammatory responses, including those seen in allergic diseases (Wang et al., 2023). Activation of NF-κB depends on the degradation of IκB (Liu et al., 2017). Following this, NF-κB is transported to the nucleus and stimulates the upregulation of several crucial inflammatory genes that encode IL-6, IL-1β, and TNF-α, thus promoting inflammation (Wang and Hauenstein, 2020). To investigate the potential inhibitory effect of GLE on the activation of MAPKs and NF-κB, we conducted western blots on nuclear and cytoplasmic extracts. Our findings indicate that GLE significantly decreased the phosphorylation of ERK1/2, JNK, and p38 induced by IgE/DNP-BSA (Figures 5A, B). NF-κB (p65) expression, which was enhanced in the nucleus by IgE/DNP-BSA stimulation of RBL-2H3 cells, was decreased by GLE treatment in a dose-dependent manner. Moreover, IgE/DNP-BSA stimulation of RBL-2H3 cells increased the expression of phosphorylated IκBα (p-IκBα) protein in the cytoplasmic fraction, which was significantly reduced by GLE treatment. The expression of inflammation-related COX-2 and TNF-α proteins was elevated by IgE/DNP-BSA stimulation of RBL-2H3 cells but reduced in a concentration-dependent manner with GLE treatment (Figures 5C, D). Our findings demonstrate that GLE inhibits MAPK phosphorylation and NF-κB translocation into the nucleus in RBL-2H3 cells activated by IgE/DNP-BSA.

### 3.5 GLE ameliorates atopic symptoms DNCB-induced AD mice-

**FIGURE 6:**
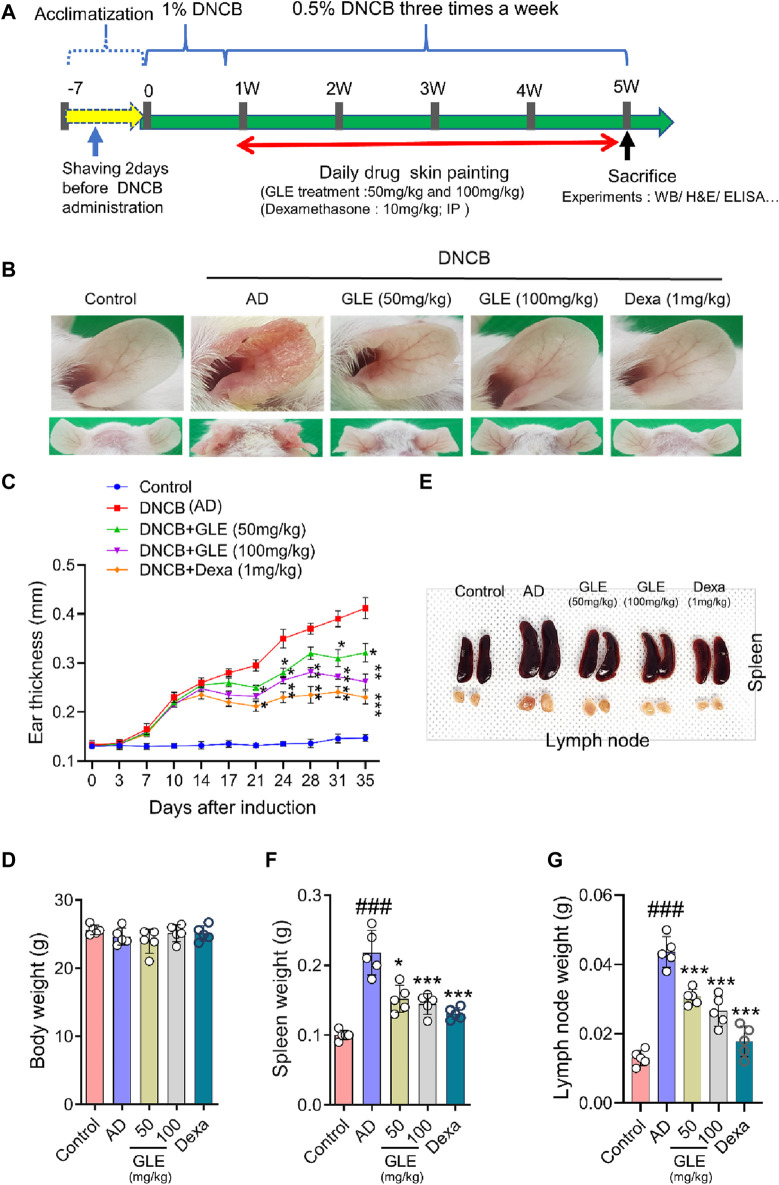
Effects of GLE on DNCB-Induced AD-Like Symptoms. **(A)** Schedule of experiment. DNCB was applied to induce AD-like symptoms in Balb/C mice. Ear skin lesions in the mouse group on the final day were photographed **(B)** and ear thickness was recorded from day 0 to day 35 with a digital indicator thickness gauge **(C)**. At the end of the experiment, body weight **(D)**, spleen, and lymph nodes in mice were and photographed **(E)** and weighed **(F, G)**. Data were expressed as mean ± SEM. ^#^ *p* < 0.05, ^##^ *p* < 0.01, and ^###^ *p* < 0.001 vs. Control; ^*^ *p* < 0.05, ^**^ *p* < 0.01, and ^***^ *p* < 0.001 vs. AD. GLE: Ginseng Leaf Extracts, AD: DNCB (2,4-dinitrochlorobenzene) treated group.

AD is characterized by xerosis (dry skin), erythema, itching, and scratching (D'Auria et al., 2016). To investigate the effect of GLE on DNCB-induced atopy-like symptoms in BALB/c mice, indicated doses of GLE (50 and 100 mg/kg) were painted on the dorsal skin and ears during the experiment (Figure 6A). Ear skin lesions were imaged (Figure 6B), and ear thickness (Figure 6C) was measured. Ear thickness, severe erythema (redness), and keratosis (thickened skin) continued to increase in the DNCB-treated group compared to the control group, but these symptoms were significantly alleviated by applying GLE (Figure 6B). Treatment with GLE at 50 and 100 mg/kg significantly attenuated the increase in ear thickness (Figure 6C). However, there was no significant difference in attenuation between the two concentrations, as depicted in Figures 6B, C. Throughout the experiment, consistent GLE administration did not significantly alter the mice’s body weights (Figure 6D) or liver weights (data not presented), implying that GLE did not exhibit any toxicity. To investigated the effect of DNCB-induced skin inflammation on other organs, submandibular lymph nodes and spleens were collected. Figures 6E–G show a significant increase in the weight of the DNCB-treated group’s (AD) lymph nodes and spleens compared to the control group. However, treatment with GLE at 50 and 100 mg/kg resulted in lighter lymph nodes and spleens than the AD group. The data indicate that administration of 50 and 100 mg/kg of GLE was not harmful and improved symptoms of atopic reactions and inflammation caused by DNCB.

### 3.6 GLE inhibits the infiltration of mast cells in DNCB-induced AD mice

**FIGURE 7:**
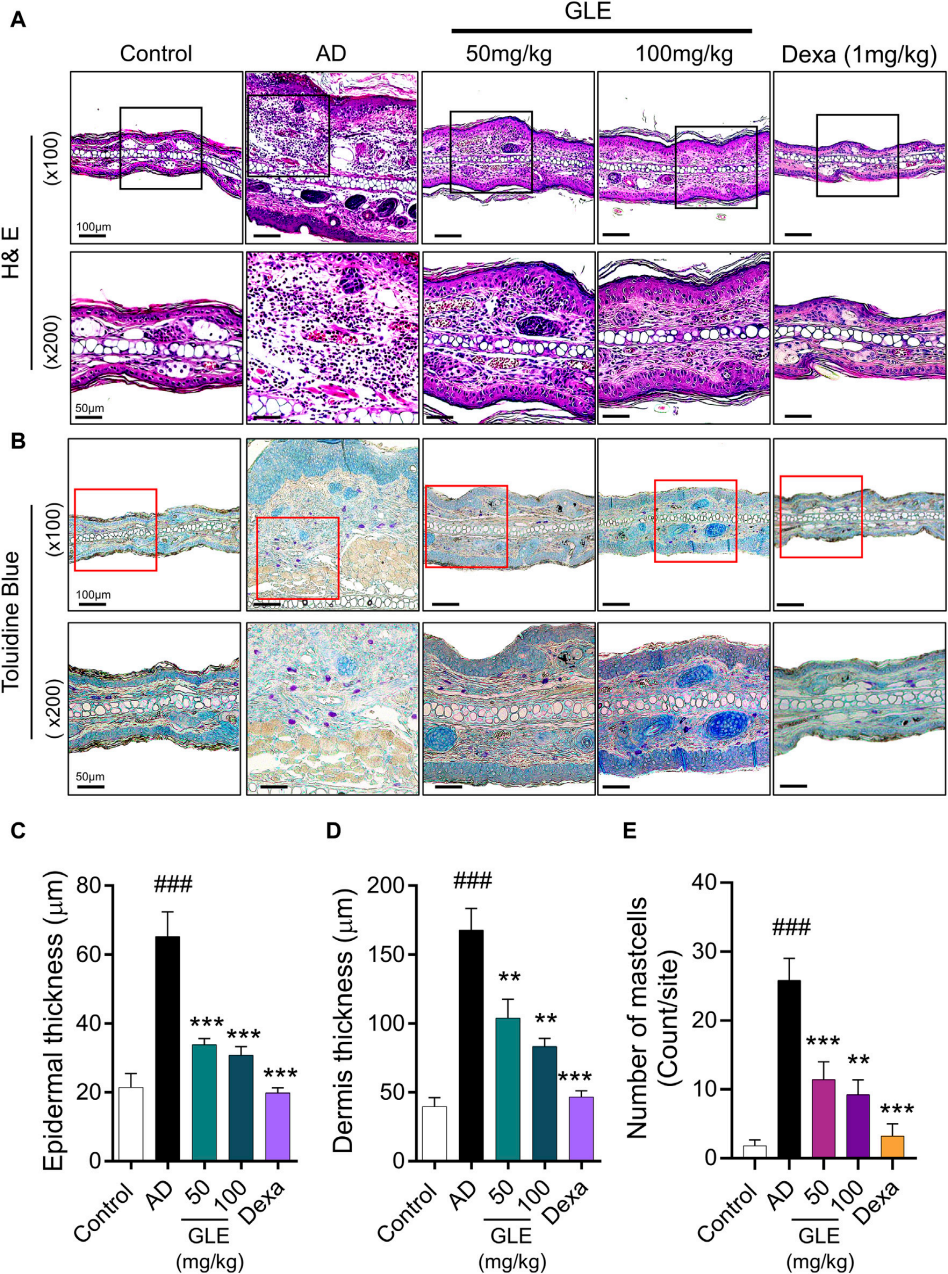
Effect of GLE on histological features in an AD-induced mice. **(A, B)** H&E and Toluidine blue (TB) stained ear skin on the last day of the experiment. Representative photomicrographs of H&E and TB staining of mouse ear skin tissue. The thickness of the epidermis **(C)** and dermis **(D)** was analyzed using H&E staining, and the count of mast cells **(E)** was determined through TB staining. Data were expressed as mean ± SEM ^###^ *p* < 0.001 vs. Control; ^***^ *p* < 0.001 vs. AD. H&E: Hematoxylin & Eosin, GLE: Ginseng Leaf Extracts, AD: DNCB-treated group.

Classic manifestations of AD, such as hyperkeratosis, severe erythema, and dryness accompanied by inflammatory cell infiltration, were observed in the mice subjected to DNCB application. To evaluate the effect of GLE on histologic changes in DNCB-induced AD-like dermatitis, epidermal/dermal thickness and mast cell infiltration were examined by staining with H&E and toluidine blue (TB). The epidermis and dermis thickness of the ear tissue of DNCB-treated mice was thicker than that of the control. GLE treatment significantly thinned the ear skin tissue as shown in Figures 7A–D. GLE also significantly lowered the elevated levels of mast cell infiltration in the dermis of DNCB-induced atopic mice (Figures 7E, F). These findings suggest that administering GLE to DNCB-treated mice blocks the infiltration of mast cells in atopic skin lesions.

### 3.7 GLE inhibits the secretion of pro-inflammatory cytokines and inflammatory mediators in DNCB-induced AD mice

**FIGURE 8:**
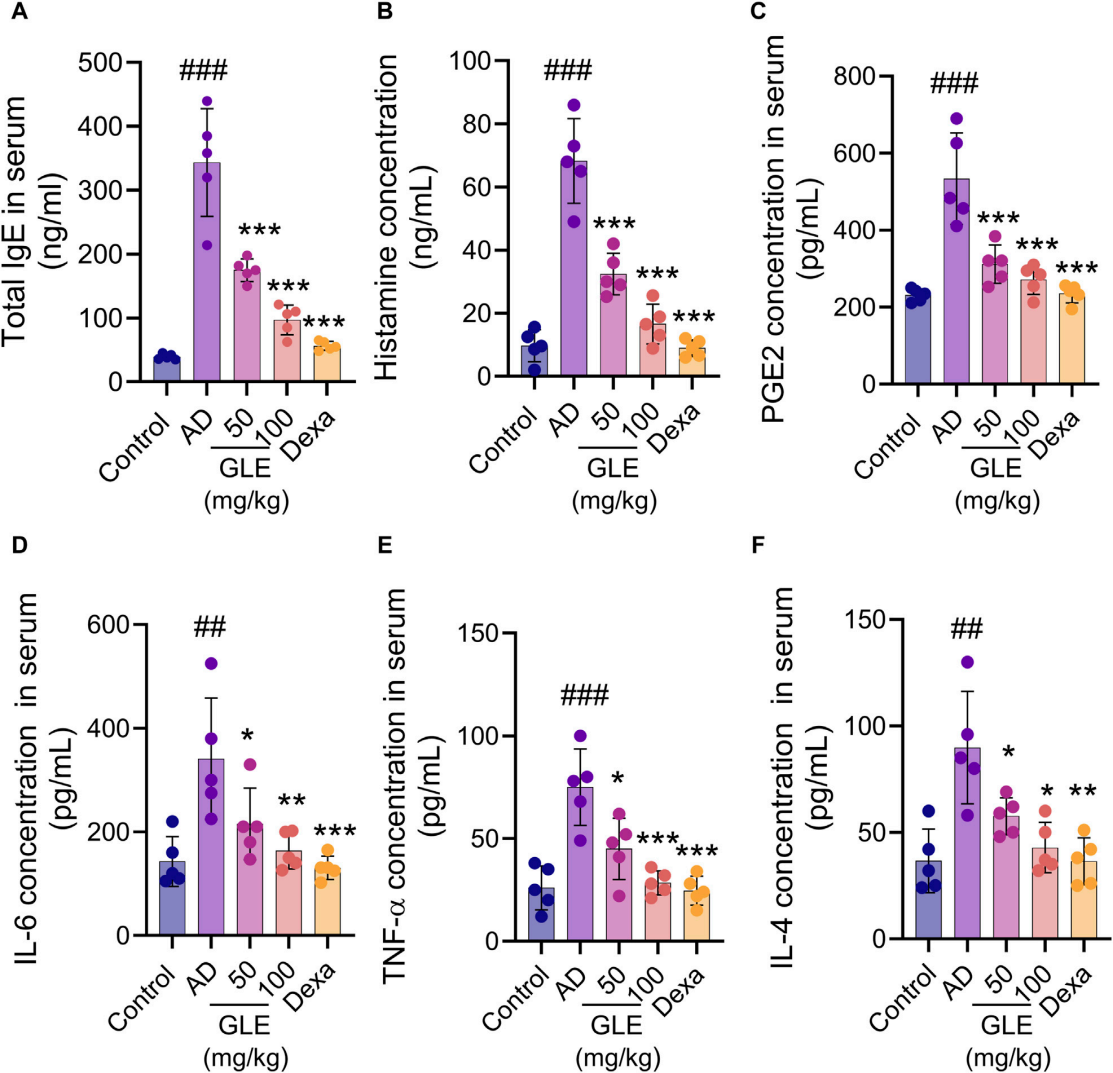
Impact of GLE on cytokine and AD-mediator levels in the serum of mice with AD-induced condition. Serum samples were collected from mice on day 35 of induction and analyzed for levels of total IgE **(A)**, Histamine **(B)**, PGE2 **(C)**, IL-6 **(D)**, TNF-α **(E)**, and IL-4 **(F)** using ELISA. Data were expressed as mean ± SEM. ^###^ *p* < 0.001 vs. Control; *p < 0.05, **p < 0.01, and ***p < 0.001 vs. AD. IgE: Immunoglobulin E, PGE2: Prostaglandin E, IL: Interleukin, TNF-α: tumor necrosis factor α, GLE: Ginseng Leaf Extracts, AD: DNCB-treated group.

The hyperproduction of IgE is characteristic of allergic hypersensitivity and an indicator of the magnitude of the allergic immune responses in the development of AD (Park et al., 2018). Mast cells activated in AD secrete a range of substances, including growth factors, cytokines (IL-4, IL-5, IL-16, and IL-13), chemokines, protein mediators (histamine), and lipid mediators (prostaglandins and leukotrienes). These substances result in the activation of Th2 cells, triggering the skin’s inflammatory response and subsequent itching (Kawakami et al., 2009). To investigate the effect of GLE on atopy-related mediators, the concentrations of IgE, histamine, PGE2, IL-4, IL-6, and TNF-α in the mice’s serum were measured using the ELISA method. GLE treatment lessened the increase in serum IgE concentration caused by repeated topical application of DNCB (Figure 8A). The serum levels of histamine, PGE2, IL-6, TNF-α, and IL-4 were significantly higher in the group treated with DNCB compared to the control group. GLE treatment reduced this increase (Figures 8B–F). These results demonstrate that GLE can effectively reduce serum IgE levels and inhibit the release of inflammatory cytokines in DNCB-induced AD mice.

### 3.8 GLE regulates the expression of inflammasome markers in DNCB-induced AD mice

**FIGURE 9:**
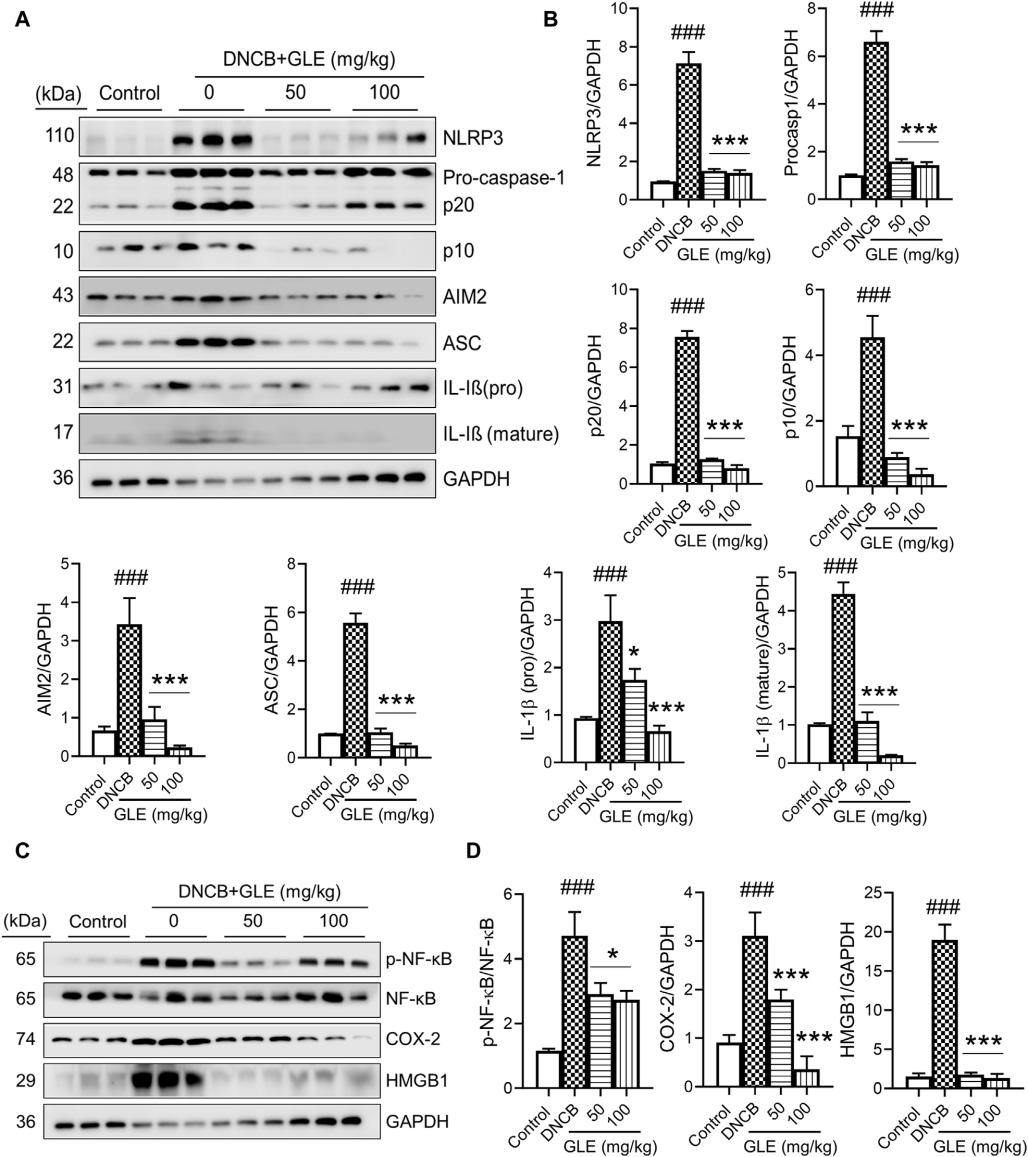
Effect of GLE on activation of inflammasome and NF-κB signaling pathway in ear skin lesions of AD-induced mice. **(A, C)** At the end of the study, Western blotting was performed to measure inflammasome components, NF-κB, p-NF-κB, HMGB1, and COX-2 in the skin lesions of the ear. **(B–D)** Protein levels were quantified by densitometry with Image J program. Data were expressed as mean ± SD. ^###^ *p* < 0.001 vs. Control; ^**^ *p* < 0.05 ^***^ *p* < 0.001 vs. DNCB. NLRP3: NOD-, LRR- and pyrin domain-containing protein 3, AIM2: Absent In Melanoma 2, ASC: Apoptosis-associated speck like protein containing a caspase recruitment domain, IL: Interleukin, NF- κB: Nuclear factor kappa B, COX-2: Cyclooxygenase-2, HMGB1: High mobility group box 1.

Recently, studies have found that the overactivation of NLRP3 (NOD-like receptor) inflammasomes is a significant factor in inflammatory skin conditions like AD (Niebuhr et al., 2014). Furthermore, the activation of NF-κB contributes to the activation of inflammatory cytokines, such as TNF-α, IL-1ß, and IL-6, which are essential indicators of inflammatory diseases (Liu et al., 2017). We conducted Western blot analysis to examine the impact of GLE on inflammasome and NF-κB activation in mice with DNCB-induced AD. The topical application of GLE significantly reduced protein levels of NLRP3, AIM2, ASC, pro-caspase-1, and cleaved casp-1 (p20, p10), which were elevated in the AD group. Additionally, the protein expression of IL-1β (mature) and HMGB1, were also significantly decreased after GLE treatment (Figures 9A–D). NF-κB activation occurs through IκB degradation (Liu et al., 2017) and results in the transcription of NLRP3 and other crucial inflammatory genes by the activated NF-κB (Wang and Hauenstein, 2020). GLE treatment inhibited the activation of NF-κB and expression of the COX-2 protein induced by DNCB (Figures 9C, D). Overall, GLE treatment improved skin lesion inflammation in DNCB-induced AD mice by inhibiting inflammasome and NF-κB activation.

## 4 Discussion

In East Asia, ginseng has been utilized as a traditional herbal medicine for thousands of years. Its major pharmacological properties, including anti-cancer, immunomodulatory, antioxidant, anti-inflammatory, and neuroprotective actions, have been attributed to ginsenosides (Shin et al., 2002). It was used medicinally in ancient China for inflammatory skin diseases, such as AD (Kimura et al., 2012). Traditionally, the roots were used, but research has shown that other parts of the ginseng plant, including the stem, leaves and berries, contain over 60 ginsenosides (Hou, 1977; Dey et al., 2003; Zhang et al., 2008; Wang et al., 2009). In the American ginseng plant, total ginsenoside concentrations are higher in the leaves than in the berries or roots (Xie et al., 2004). GLE exhibits pharmacological activity against type II diabetes, improves central nervous system symptoms, and has various effects to prevent aging, cancer, inflammation, obesity, and oxidation (Niebuhr et al., 2014). However, compared to ginseng roots, functional studies of GLE are still lacking, and the effects of GLE on allergy-related mechanisms still need to be elucidated. This study demonstrated that GLE inhibits allergen-induced mast cell activity in RBL-2H3 cells and improves DNCB-induced inflammatory skin disease. To the best of our knowledge, this is the first report to demonstrate that GLE regulates mast cell activity and inflammatory skin diseases.

There is a clear difference in the ginsenoside content of ginseng leaves (Wang et al., 2009), and HPLC results confirmed that ginsenosides such as Rb3, Rd, Re, Rb2, RC, Rb1, and Rg1 are the main components of GLE under our conditions. Rb3 attenuated LPS-mediated inflammation in macrophages (Xu et al., 2022). Ginsenosides Rg and Rd inhibit IL-4 expression (Kim HI. et al., 2019), and Rb1, Rd, F2, and CK inhibit degranulation in mast cell and RBL-2H3 cells (Wang et al., 2017). In this context, the current manuscript shows that the active components of GLE, including Rb3, Rd, and Re, can inhibit mast cell activity and allergic inflammatory responses.

The pathogenesis of AD is an inflammatory reaction caused by imbalanced immune mechanisms, defective skin barriers, and oxidative stress (D'Auria et al., 2016; Ji and Li, 2016). Mast cells, pivotal to the pathogenesis of allergic inflammation, are implicated in both innate and acquired immunity and are recognized for their involvement in regulatory processes (Krystel-Whittemore et al., 2015). The cross-linking of allergen-specific IgE in mast cells activates the high-affinity membrane-bound receptor FcεRI, initiating intracellular signaling cascades and inducing allergic reactions through degranulation and the release of inflammatory mediators. RBL-2H3 cells express high levels of FcεRI and are commonly used to assess whether IgE is binding to the FcεRI receptor and to determine subsequent downstream events (Passante and Frankish, 2009). We therefore selected these cells as a suitable model to validate GLE’s anti-allergic inflammatory activity. Ginsenoside Rg3 reduces the release of histamine and the production of inflammatory cytokines, such as IL-1ß, IL-6, and TNF-α (Kee and Hong, 2019). Red ginseng (RG) inhibits chronic airway inflammation and reduces mast cell populations in ovalbumin-sensitized mice (Babayigit et al., 2008). In our study, GLE demonstrated an inhibitory effect on β-hexosaminidase and histamine release in IgE/anti-DNP BSA stimulated RBL-2H3 cells. This suggests that GLE itself has a significant degranulation inhibitory effect and is useful for developing new strategies for allergy treatment.

Mast cell granules contain substances that are stored within the granule prior to stimulation (e.g., histamine, proteoglycan, TNF-α) and newly synthesized mediators that are produced and released following stimulation [e.g., prostaglandins (PG) and leukotrienes (LTs)] (Kleij and Bienenstock, 2005). The primary PG produced by mast cells is PGD2, with PGE2 and LTB4 also present in high concentrations (Fogh et al., 1989; Jin et al., 2009). The roles of LT and PG in allergic inflammatory responses such as asthma and atopy are well established. The skin of patients with AD exhibits high concentrations of eicosanoids (Jin et al., 2009), and LTB4 is involved in acute inflammation in human and mouse models of AD. CysLT is associated with chronic AD, which is characterized by collagen deposition, skin thickening, and fibrosis (Oyoshi et al., 2012). In our experiments, we observed that the expression and secretion of PGE2 and LTC4 were suppressed in activated mast cells. We also showed that GLE treatment lowered PGE2 levels in mice with skin inflammation. This suggests that GLE is effective in improving inflammation symptoms.

Mast cells are involved in both early and late allergic reactions. In a late allergic reactions, cytokines such as (IL)-4, IL-5, IL-6, IL-8, IL-13, and TNF-α are produced and released (Kleij and Bienenstock, 2005). The release of cytokines from activated mast cells represents a significant mediator of allergic and inflammatory diseases. The production of cytokines in activated mast cells is the result of induced transcription of cytokine genes. The transcription factor NF-κB is crucial in initiating inflammatory responses because it regulates the genes encoding adhesion molecules, chemokines, inducers (such as COX-2 and iNOS), and pro-inflammatory cytokines (Liu et al., 2017). Upon stimulation, NF-κB is activated through the dissociation of the inhibitory IκB protein from NF-κB dimers, leading the liberated NF-κB to translocate into the nucleus and initiate the target genes’ transcription (Chen and Chen, 2013; Liu et al., 2017). MAPKs, including ERKs, JNKs, and p38, are a group of proteins that regulate inflammatory responses and the immune system. This MAPK activity is associated with mast cell degranulation and subsequent cytokine production. Previous research has demonstrated that p38 MAPK activation can result from stress and inflammatory stimuli and that this activation is involved in pulmonary inflammation (Millan et al., 2011; Kyriakis and Avruch, 2012) The current study demonstrated that GLE inhibits the release and expression of cytokines in activated mast cells by suppressing MAPK/NF-κB pathways.

COX-2 is known to be upregulated during the inflammatory process (Zidar et al., 2009). iNOS is inhibited in most tissues but transcriptionally activated by inflammatory cytokines. The resulting product of its activity, NO, contributes to various diseases (Zamora et al., 2000). Multiple studies have provided evidence that chronic inflammatory diseases involve not only certain inducible enzymes (COX and iNOS) and cytokines but also the products generated from their activities (Zamora et al., 2000; Ji and Li, 2016). In lipopolysaccharide (LPS)-induced RAW264.7 cells, ginsenoside CK has exhibited inhibitory effects on COX-2 expression, prostaglandin E2 production, NF-κB activation, and NO production (Park et al., 2005). In macrophages, ginsenoside Rg3 inhibits inflammasome activity and downregulates the expression of NO, ROS, and iNOS (Yoon et al., 2015). Increases in allergic inflammatory mediators, inflammatory cell infiltration, and an overproduction of ROS are associated with allergic inflammation, for example, in AD and asthma (D'Auria et al., 2016; Ji and Li, 2016). Our experiments showed that GLE inhibits the production of pro-inflammatory cytokines, inflammatory mediators (LTC4 and PGE2), and ROS and NO production in IgE/DNP-BSA-induced RBL-2H3 cells. Based on our data, GLE emerges as a promising candidate to treat allergic diseases.

Ginsenosides can modulate various signaling molecules associated with inflammation and immune regulation, primarily through the MAPK and NF-κB pathways. Korean red ginseng treatment has been shown to alleviate ear thickness malformations, reduce serum IgE levels, and decrease the production of proinflammatory cytokines (IL-1ß, IL-6, and IL-8). The suppressive effect on serum IgE levels and Th2 cytokines (IL-4 and IL-10) was attributed to the inhibition of the MAPK/NF-κB pathways in both DNFB and DNCB-induced AD models (Sohn et al., 2011; Kee et al., 2017). Prior studies have shown that ginsenoside Re effectively inhibits the activation of MAPKs and transcription factors, including NF-κB and c-Fos, *in vivo* (Lee et al., 2018). Our study confirmed that GLE treatment could potentially improve mast cell-mediated inflammatory activity *via* the MAPK/NF-κB pathway. NF-κB is a central mediator of the priming signal for NLRP3 inflammasome activation (Bauernfeind et al., 2009). Therefore, we sought to confirm GLE’s effects by focusing on these pathways in the DNCB-induced AD mouse model.

Inflammasomes play a pivotal role in skin inflammatory disease, including AD (Calabrese et al., 2024). It is involved in the maturation of inflammatory cytokines, such as IL-1ß and IL-18, which are related to the innate immune response to infection or cellular stress (Chen and Nunez, 2011). The most extensively studied of these is NLRP3 inflammasome, which is composed of NLRP3, ASC, and pro-caspase-1, as well as an essential regulatory protein, NIMA-related kinase 7 (Broz and Dixit, 2016). NLRP3 inflammasome activation has been implicated in a variety of inflammatory and autoimmune conditions, including metabolic diseases, such as type 2 diabetes, arteriosclerosis, and obesity, as well as neurodegenerative diseases, such as Alzheimer’s and Parkinson’s disease (Strowig et al., 2012). In addition, this activation reportedly triggers allergic inflammation, including AD (Niebuhr et al., 2014; Herberth et al., 2015). Korean red ginseng extract inhibits the activation of NLRP3 and AIM2 inflammasomes during macrophage-mediated inflammatory response (Kim et al., 2014).

Our study indicates that GLE treatment significantly lowered ear thickness and reduced the weight of the spleen and lymph nodes in DNCB-induced AD mice. GLE significantly reduced mast cell infiltration in the skin epidermis and dermis, as well as serum levels of IgE, histamine, PGE2, and pro-inflammatory cytokines IL-4, IL-6, and TNF-α. In addition, the expression of NLRP3, ASC, AIM2, mature casp-1, and mature IL-1β and p-NF-κB induced by DNCB application was downregulated by GLE treatment.

These results indicate that GLE treatment alleviates atopic symptoms by suppressing the production of IgE as well as various inflammatory cytokines and mediators, and the signaling pathways associated with NF-κB and NLRP3 inflammasomes. Collectively, these data demonstrate that GLE’s inhibition of the inflammasome and NF-κB signaling pathways may be an important aspect of treating allergic inflammation.

## 5 Conclusion

The topical application of GLE to mice with DNCB-induced AD improved atopy symptoms by reducing the production of several pro-inflammatory cytokines and mediators by inhibiting the NF-κB and inflammasome pathways. GLE also decreased the production and expression of ROS, inflammatory cytokines, and mediators in RBL-2H3 cells stimulated by IgE/DNP-BSA. GLE administration has important effects on the mechanisms involved in the resolution of allergic inflammation. We propose that GLE is a reliable and efficient pharmacological intervention that offers both safety and efficacy for the treatment of AD.

## Data Availability

The original contributions presented in the study are included in the article/Supplementary Material, further inquiries can be directed to the corresponding author.

## Abbreviations

GLE, ginseng leaf extract; AD, Atopic dermatitis; BSA, Bovine serum albumin; COX, Cyclooxygenase; DMEM, Dulbecco’s modified Eagle’s medium; DMSO, Dimethyl sulfoxide; DNCB, 2,4-dinitrochlorobenzene; ELISA, Enzyme-linked immunosorbent assay; FBS, Fetal bovine serum; H&E, Hematoxylin and eosin; IL, Interleukin; iNOS, inducible nitric oxide synthase; MAPK, mitogen-activated protein kinase; MTT, 3-(4,5-dimethylthiazol-2-yl)-2,5 diphenyltetrazolium bromide; NF-κB, Nuclear factor-kappa B; NO, Nitric oxide; PBS, Phosphate-buffered saline; TB, Toluidine blue; TNF-α, tumor necrosis factor; HEPES, 4-(2-hydroxyethyl)-1-piperazineethanesulfonic acid; NLR, nucleotide oligomerization domain-like receptor; ROS, reactive oxygen species; Dexa, dexamethasone.
