# Supplementary figures and images for: Therapeutic potential of ginseng leaf extract in inhibiting mast cell-mediated allergic inflammation and atopic dermatitis-like skin inflammation in DNCB-treated mice
Source: Front Pharmacol. 2024 May 22;15:1403285. doi: 10.3389/fphar.2024.1403285 (PMC11150533; doi:10.3389/fphar.2024.1403285)

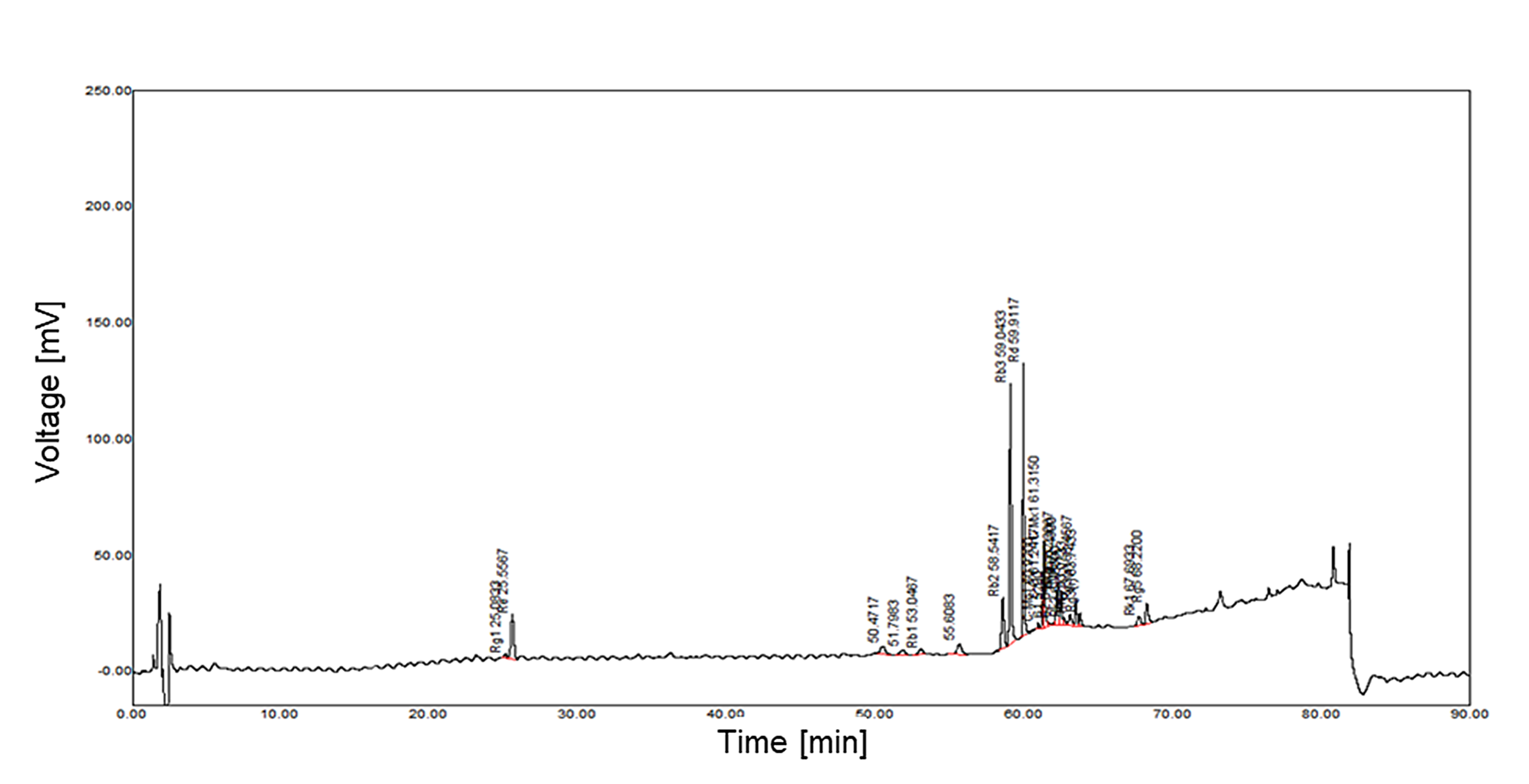

Supplement: Supplementary file 1 [file Image1.tif]
